# Association Between Psychosocial Factors and the Need for Orthodontic Treatment Based on Self-Perception

**DOI:** 10.3390/jcm15041347

**Published:** 2026-02-09

**Authors:** Olimpia Bunta, Diana Petca, Gabriela Ofelia Chiciudean, Dan Sancraian, Daniel Ioan Chiciudean, Dana Festila

**Affiliations:** 1 Orthodontics Department, Faculty of Dental Medicine, Iuliu Hatieganu University of Medicine and Pharmacy, 400012 Cluj-Napoca, Romania; 2 Economic Sciences, Faculty of Horticulture and Business in Rural Development, University of Agricultural Sciences and Veterinary Medicine, 400372 Cluj-Napoca, Romania; daniel.chiciudean@usamvcluj.ro

**Keywords:** perception of smile, self-esteem, orthodontic treatment, malocclusion

## Abstract

**Introduction:** Smile esthetics play a central role in social interaction, self-esteem, and self-confidence, and may influence individuals’ perception of orthodontic treatment need. Understanding how patients evaluate their own smile and how this perception relates to treatment demand is increasingly relevant for patient-centered orthodontic care. This study aimed to examine the associations between demographic factors, self-perception of smile esthetics, psychosocial behaviors, and perceived need for orthodontic treatment. **Materials and Methods:** A cross-sectional study was carried out using a questionnaire, yielding 303 valid responses. The questionnaire assessed demographic characteristics, satisfaction with smile appearance, smile-related avoidance behaviors, awareness of dental esthetics, and self-perceived orthodontic treatment need. Associations between variables were analyzed using Chi-square tests, Kendall’s tau_b, Spearman’s rho, and Cramer’s V coefficients. Multinomial logistic regression was performed to identify factors associated with perceived treatment need. **Results:** Gender was significantly associated with satisfaction with smile appearance, concern about dental esthetics, and awareness of the importance of aligned teeth (*p* ≤ 0.001), with weak to moderate effect sizes. Age showed statistically significant but weak correlations with smile-related behaviors and perceived treatment need (|τ| ≈ 0.11–0.12; *p* < 0.05). Lower satisfaction with one’s smile was moderately associated with a higher perceived need for orthodontic treatment (τb = −0.20; *p* < 0.001). Avoidance of smiling and covering the mouth while speaking were positively associated with perceived treatment need (*p* < 0.01). **Discussions:** Regression analysis confirmed that dissatisfaction with smile esthetics, smile-avoidance behaviors, and belief in the esthetic benefits of dental alignment were significant predictors of perceived orthodontic treatment need. Self-perception of smile esthetics and related psychosocial behaviors are significantly associated with individuals’ perceived need for orthodontic treatment. **Conclusions:** These findings underscore the importance of incorporating patient-reported perceptions and psychosocial factors into orthodontic assessment and shared decision-making.

## 1. Introduction

Nonverbal communication represents the most frequent and perceptible form of expression. People frequently convey their emotions, either consciously or unconsciously, through nonverbal cues such as facial expressions and gestures [1,2]. Smiling is the most frequent facial expression. A smile represents a statement of well-being, communicates politeness and exudes self-confidence, often generating favorable social reactions or responses on the positive scale. Faces displaying a smile are generally perceived as more attractive than non-smiling faces [1,3].

One of the primary reasons patients pursue orthodontic treatment is to improve the esthetic appearance of their smile [4]. The appearance of the smile and its perception play a significant role in an individual’s social and emotional functioning [5]. A favorable perception of one’s smile is frequently associated with elevated self-esteem and enhanced self-confidence [6,7].

Despite widespread agreement that orthodontic treatment enhances the esthetics of the smile, its specific impact on perceived smile attractiveness is not yet well established. Perceiving the esthetics of a smile is a cognitive process, influenced by established criteria of symmetry, facial harmony and beauty [8,9]. Even minor deviations from commonly accepted esthetic standards can particularly impact young adults, potentially lowering their self-esteem and fostering a sense of inferiority compared to their peers. This diminished self-confidence is significant, as it frequently has a negative effect on an individual’s overall quality of life [10,11].

Malocclusion should not be perceived as a disease, but rather a deviation in the esthetics of what is considered normal and/or acceptable in society [12,13]. The need for orthodontic treatment is either reported subjectively by the patient or their legal guardian, or measured objectively by the dentist or orthodontist. However, inconsistencies related to personal characteristics and/or environmental factors have been reported between objective and subjective assessments of orthodontic treatment need [14].

The present study is grounded in a psychosocial framework of dental esthetics, which posits that self-perceived smile appearance influences emotional responses, self-esteem, and social behaviors, ultimately shaping health-related perceptions and decision-making. Within this framework, dental esthetics are not evaluated solely through objective clinical indicators but through subjective self-assessment, which mediates the psychosocial impact of malocclusion. Accordingly, psychosocial constructs such as satisfaction with one’s smile, avoidance behaviors, and awareness of dental esthetics were operationalized through self-reported questionnaire items adapted from established instruments, while perceived orthodontic treatment need was assessed as a subjective outcome reflecting individual appraisal rather than clinical diagnosis.

The main purpose of this study is to examine the associations between demographic factors, self-perception of smile esthetics, psychosocial behaviors, and perceived need for orthodontic treatment.

Hypotheses

Based on existing literature, the present study tested the following hypotheses:

**Hypotheses** **1.** *Demographic variables (age, gender, and background) are significantly associated with self-perception of smile esthetics and satisfaction*.

**Hypotheses** **2.** *Lower satisfaction with one’s smile and the presence of smile-avoidance behaviors are associated with a higher perceived need for orthodontic treatment*.

**Hypotheses** **3.** *Greater awareness of the importance of aligned teeth is associated with an increased likelihood of perceiving orthodontic treatment as necessary*.

## 2. Materials and Methods

This cross-sectional study was conducted during the month of December 2023 using a self-administered online questionnaire created with Google Forms (Google LLC, Mountain View, CA, USA). Participation was voluntary and anonymous. Inclusion criteria were age ≥10 years, owning a smart mobile phone, ability to understand and complete the online questionnaire, having had only one initial orthodontic consult and existence of informed consent prior to participation. Exclusion criteria included patients with more than one orthodontic consult and incomplete questionnaires. As all recipients of the questionnaire have had an initial orthodontic consult in the Orthodontics Department of the “Iuliu Hatieganu” University of Medicine and Pharmacy, Cluj-Napoca, Romania, they had signed or had their legal guardians sign the Department’s informed consent form, expressing their willingness to participate in anonymous surveys, without having any identifiable personal or sensitive data collected. The Orthodontics Department informed consent has been approved by the Ethics Committee (approval no. 20/20.01.2014).

The questionnaire was electronically disseminated using the WhatsApp messaging service (WhatsApp Ireland Limited, Dublin, Ireland) to a total of 385 possible respondents that have met the inclusion criteria for this study. The final sample size was determined by the number of complete responses obtained during the data collection period. A total of 303 valid questionnaires were included in the analysis. The absence of formal sample size calculation was acknowledged as a limitation with potential implications for statistical power and generalizability.

The questionnaire consisted of 11 mandatory items grouped into three domains:

(1) demographic data (age, gender, background),

(2) self-perception and psychosocial aspects related to smile esthetics, and

(3) knowledge and awareness regarding the importance of dental alignment and perceived need for orthodontic treatment. Items assessing self-perception and psychosocial impact were adapted from internationally validated instruments, including the Rosenberg Self-Esteem Scale [15,16], the Overall Anxiety Severity and Impairment Scale (OASIS) [17], and the Oral Health Impact Profile (OHIP) [18] (Table S1)**.** These instruments were used as conceptual references rather than as full standardized scales. No formal translation procedure, cultural adaptation, pilot testing, or internal consistency analysis (e.g., Cronbach’s α) was conducted for the adapted questionnaire. Self-perception variables included satisfaction with one’s smile, concern about dental appearance, avoidance of smiling, covering the mouth while speaking, and awareness of the impact of smiling on mood and self-confidence. Perceived orthodontic treatment need was assessed as a subjective outcome reflecting individual self-assessment rather than clinical diagnosis.

Participants were recruited through online dissemination of the questionnaire link. Although a simple probability sampling approach was intended, the use of an online survey resulted in a self-selected sample. This recruitment method may have introduced selection and nonresponse biases, particularly among individuals with limited digital access or skills, limited time availability, or discomfort related to dental appearance.

Statistical analyses were performed using IBM SPSS Statistics version 27 (IBM Corp., Armonk, NY, USA). A conventional alpha error level of 0.05 was applied for all statistical tests. Descriptive statistics were used to summarize the study sample, with categorical variables including age, gender, and background presented as absolute frequencies and percentages. Associations between categorical variables were assessed using the Chi-square test, with a *p*-value < 0.05 considered statistically significant. Measures of association, including Phi and Cramer’s V coefficients, were used to estimate the strength of statistically significant relationships. Ordinal variables were analyzed using Kendall’s tau_b and Spearman’s rho correlation coefficients, selected based on the ordinal nature of questionnaire responses and distributional characteristics. Variable categorization followed the predefined questionnaire response options and reflected increasing levels of satisfaction, awareness, or frequency of behaviors. Multinomial logistic regression analysis was performed to explore factors associated with perceived orthodontic treatment need, categorized as “yes,” “no,” or “I do not know.” Results were expressed as relative likelihoods to describe the association between psychosocial factors and perceived treatment need.

## 3. Results

The total number of respondents for this survey was 303.

### 3.1. Descriptive Analysis

#### 3.1.1. Respondents’ Personal Data

The vast majority of respondents (64%) are females, live in urban areas (68%), are between 21 and 35 years old (40%), followed by adults over 35 years old (38%), and are from an urban background (Table 1).

#### 3.1.2. Patient Self-Perception

Data show that the majority of respondents are satisfied with the appearance of their smile (56%), but the statistical results of the respondents show that 31% of them sometimes avoided smiling because of the appearance of their teeth, and 19% of them covered their mouth when speaking (Table 2).

**Figure 1:**
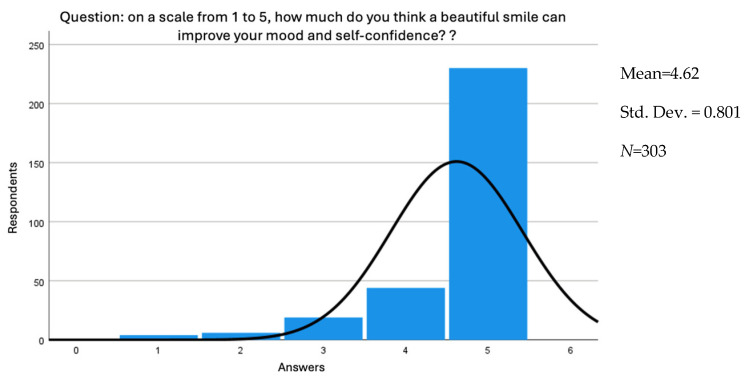
Smile awareness histogram.

Regarding awareness of the impact of smiling on mood and self-confidence, the histogram, mean, standard deviation, and normal distribution curve can be observed in Figure 1. The mean awareness is 4.62, and the standard deviation is 0.801, meaning that, on average, a respondent’s awareness varies by this value (Figure 1).

#### 3.1.3. Knowledge and Awareness of the Need for and Importance of Orthodontic Treatment

The results show that 87% of respondents believe that aligned teeth are important for facial appearance, 78% believe that their smile would be more beautiful if they had their teeth aligned, and 42% believe they need orthodontic treatment, while 33% do not believe this.

### 3.2. Bivariate Analysis

#### 3.2.1. Respondents’ Perception and Awareness of the Need for Orthodontic Treatment According to Demographic Data

This study intended to find the relationship between demographic characteristics (such as age, gender, background) and perceptions of smiles, teeth, and the need for orthodontic treatment. If the *p*-value is less than 0.05, it means that there is a significant link.

##### Gender Influences Perceptions of Smiles

The distribution of respondents according to gender and level of satisfaction with their smile indicated a higher proportion of female respondents that reported being satisfied or very satisfied with their smile compared to male respondents. In contrast, male respondents more frequently reported indifferent or dissatisfied levels of satisfaction. These distributions suggest noticeable differences in self-reported smile satisfaction between genders, which were further examined using inferential statistical analysis.

Table 3 summarizes the associations between gender and smile-related perceptions and awareness, as assessed using the Chi-square test. A statistically significant association was observed between gender and the level of satisfaction with one’s smile (χ^2^ = 19.532, df = 4, *p* = 0.001), indicating that females reported higher levels of satisfaction compared to males. The strength of this association was weak to moderate, as indicated by Cramer’s V values ranging between 0.25 and 0.30.

A significant association was also identified between gender and concern about the appearance of teeth (χ^2^ = 25.639, df = 2, *p* < 0.001), with females expressing greater concern regarding dental esthetics. The effect size for this association (Phi and Cramer’s V = 0.291) suggests a moderate relationship.

Additionally, gender was significantly associated with awareness of the importance of straight teeth for facial appearance (*p* < 0.05), with females demonstrating higher levels of awareness than males. Overall, these findings indicate that gender plays a statistically significant role in satisfaction with smile appearance, concern about dental esthetics, and awareness of the esthetic importance of aligned teeth, although the observed associations were of weak to moderate magnitude.

##### Age Has a Weak but Noticeable Influence on Perception

Age showed statistically significant but weak correlations with several perception-related variables. Positive correlations were observed between age and awareness that smiling improves mood (Kendall’s τ = 0.109; Spearman’s ρ = 0.120, *p* < 0.05), and between age and awareness of the importance of straight teeth (τ = 0.120; ρ = 0.134, *p* < 0.05). Negative correlations were found between age and covering the mouth when speaking (τ = −0.108; ρ = −0.116, *p* < 0.05), and between age and perceived need for orthodontic treatment (τ = −0.111; ρ = −0.122, *p* < 0.05). All associations were statistically significant but of weak magnitude (Table 4).

#### 3.2.2. Need for Treatment Based on Patient Perception and Satisfaction with Their Own Appearance

Table 5 shows the correlations between self-perception variables related to smile esthetics and the perceived need for orthodontic treatment. A statistically significant negative correlation was observed between satisfaction with one’s smile and the perceived need for treatment (Kendall’s τb = −0.200, *p* < 0.001), indicating that higher levels of satisfaction were associated with a lower likelihood of reporting a need for orthodontic treatment.

Significant positive correlations were identified between the perceived need for treatment and behaviors reflecting dissatisfaction with dental appearance, including avoidance of smiling (τb = 0.212, *p* < 0.001) and covering the mouth while speaking (τb = 0.122, *p* = 0.025). Additionally, respondents who believed their smile would be more attractive with straighter teeth were more likely to report a need for orthodontic treatment (τb = 0.154, *p* = 0.004). Concern about the appearance of teeth was also significantly associated with perceived treatment need, although with a weaker correlation (τb = −0.129, *p* = 0.017).

All observed correlations were weak to moderate in strength but statistically significant, highlighting consistent associations between self-perceived dental esthetics and the subjective need for orthodontic treatment.

Satisfaction with one’s smile was negatively associated with perceived orthodontic treatment need (Kendall’s τ = −0.200, *p* < 0.001), indicating a small-to-moderate inverse association. Avoiding smiling because of dental appearance showed a positive association with perceived treatment need (τ = 0.212, *p* < 0.001), while covering the mouth when speaking was weakly associated (τ = 0.122, *p* = 0.025). Belief that one’s smile would be more attractive with straightened teeth was also associated with perceived need (τ = 0.154, *p* = 0.004). In other words, people who feel good about themselves do not consider that they need dental work (significant negative correlations). People who avoid smiling or cover their mouths when they talk because they are ashamed of their teeth are more likely to feel they need orthodontic treatment. If a person believes that their smile would look better with straighter teeth, they are more likely to believe they need orthodontic treatment.

#### 3.2.3. Factors Associated with Perceived Orthodontic Treatment Need

A multinomial logistic regression analysis was conducted to identify factors associated with respondents’ perceived need for orthodontic treatment. The outcome variable was categorized as “Yes,” “No,” and “I do not know,” with “I do not know” used as the reference category. Predictor variables included satisfaction with one’s smile, avoidance of smiling due to dental appearance, covering the mouth while speaking, belief that the smile would be more attractive with straightened teeth, and awareness of the importance of dental alignment (Table 6).

Lower satisfaction with one’s smile was significantly associated with a higher perceived need for orthodontic treatment. Respondents reporting lower satisfaction were more likely to indicate a perceived need for treatment, whereas those with higher satisfaction were more likely to report that they did not need orthodontic treatment (*p* < 0.001).

Smile-avoidance behaviors showed consistent associations with perceived treatment need. More frequent avoidance of smiling because of dental appearance was associated with a higher perceived need for orthodontic treatment, with a moderate strength of association (*p* < 0.001). Covering the mouth while speaking was also associated with a higher perceived need for treatment, although the strength of this association was weaker (*p* < 0.05).

Belief-related variables were likewise significantly associated with perceived orthodontic treatment need. Respondents who believed that their smile would be more attractive if their teeth were straightened were more likely to report a perceived need for orthodontic treatment, showing a moderate association (*p* < 0.01). In contrast, those who did not hold this belief were more likely to report no perceived need for treatment.

Awareness of the importance of straight teeth for facial appearance showed a statistically significant but weak association with perceived treatment need. Higher awareness was associated with a greater likelihood of reporting a perceived need for orthodontic treatment, while lower awareness was associated with reporting no perceived need (*p* < 0.05).

Overall, the regression analysis demonstrated that subjective satisfaction with one’s smile, smile-related avoidance behaviors, belief in the esthetic benefits of dental alignment, and awareness of dental esthetics were significantly associated with perceived orthodontic treatment need. The observed associations ranged from weak to moderate in strength and reflect psychosocial patterns within the study population rather than causal relationships.

## 4. Discussion

The increasing demand for orthodontic treatment in adults has been attributed to multiple factors, including greater emphasis on dental esthetics, improved access to information, technological advances, and psychosocial considerations [19]. However, the present study does not aim to establish causal pathways; rather, it explores statistical associations between demographic characteristics, self-perception of smile, and perceived need for orthodontic treatment within a cross-sectional design.

Consistent with previous literature [20,21], the results indicate statistically significant associations between gender and several perception-related variables. Women reported higher levels of satisfaction with their smile and greater concern about dental appearance compared to men. These associations were statistically significant, with Cramer’s V values ranging between 0.25 and 0.30, indicating small to moderate effect sizes. Although these effects are not large, they suggest that gender is meaningfully associated with differences in smile perception and awareness of dental esthetics.

Age was also associated with several perception variables, including awareness of the importance of straight teeth and behavioral indicators such as avoiding smiling or covering the mouth when speaking. However, the correlation coefficients (Kendall’s tau and Spearman’s rho ranging from approximately 0.10 to 0.13) indicate weak associations. These findings suggest that age-related differences exist but account for only a small proportion of variance in perception and behavior. Our findings are consistent with other studies in the literature [20,22,23].

Background (urban vs. rural) showed minimal differences in self-perception of dentition, although urban respondents reported a higher perceived need for orthodontic treatment. This pattern may reflect contextual differences in access to dental services and exposure to esthetic norms; however, such interpretations remain speculative and cannot be confirmed within the present study design [24].

Regarding perceived need for orthodontic treatment, several perception-related variables were significantly associated with treatment perception. The strongest associations were observed for avoiding smiling (τ = 0.212) and satisfaction with one’s smile (τ = −0.200), both representing small to moderate effect sizes. These results indicate that individuals who report behavioral avoidance or lower satisfaction tend to report higher perceived treatment need, although these associations do not imply that dissatisfaction causes treatment demand.

Multinomial regression further showed that awareness of dental alignment, satisfaction with smile, and behavioral avoidance were significantly associated with perceived orthodontic treatment categories. The reported odds ratios (ranging approximately from 1.3 to 3.7) indicate moderate associations. For example, respondents who believed their smile would be more attractive with straighter teeth were about 2.8 times more likely to report a perceived need for orthodontic treatment. These findings should be interpreted as statistical associations within the sample rather than as evidence of behavioral prediction or causation.

Overall, the findings support existing evidence that self-perception and demographic factors are associated with perceived orthodontic treatment need [20]. However, the effect sizes are generally small to moderate, and the cross-sectional design precludes any causal inference. The results should therefore be understood as descriptive of patterns within this population rather than evidence of direct influence. Longitudinal or experimental studies would be required to clarify causal mechanisms underlying treatment-seeking behavior.

The gender effect on the demand for orthodontic treatment in this study is consistent with that reported by Roberts et al. [25], but contradicts the findings of Burden [26] and Celikel [27], who concluded that gender has no influence on the need for orthodontic treatment.

Patients in rural areas expressed a need for orthodontic treatment less frequently than those in more urban areas; they were found to have a more tolerant attitude towards malocclusions than subjects in urban areas with a high frequency of orthodontic treatment. These findings are consistent with those of Espeland et al. [24], Bergström et al. [28], and Kerosuo et al. [29].

Several limitations of the present study should be acknowledged. First, the sample size was relatively limited, and although it was sufficient to detect statistically significant associations, a larger sample could provide greater statistical power and improve the generalizability of the results. Additionally, the cross-sectional design does not allow for causal inferences, and responses were based on self-reported perceptions, which may be influenced by subjective bias. Although significant associations were identified between demographic characteristics, self-perception of smile and perceived orthodontic treatment need, inverse or bidirectional relationships cannot be excluded. For example, dissatisfaction with one’s smile may be associated with higher perceived treatment need, but individuals who already perceive a need for orthodontic treatment may also report greater dissatisfaction.

No a priori sample size calculation was performed, and the sample size was determined by the number of complete responses obtained during the study period. Although a conventional alpha level of 0.05 was applied for statistical testing, the absence of a formal power analysis may limit the ability to detect small effects and increases the risk of type II error.

The questionnaire included items adapted from internationally validated instruments such as the Rosenberg Self-Esteem Scale, the Overall Anxiety Severity and Impairment Scale (OASIS), and the Oral Health Impact Profile (OHIP). However, these instruments were not used in their original standardized form, and no formal translation procedure, cultural adaptation, pilot testing, or internal consistency analysis (e.g., Cronbach’s α) was conducted. The lack of local validation may therefore affect measurement reliability and should be addressed in future studies.

The study is also subject to potential selection bias, as participant recruitment was conducted through a voluntary, self-administered online survey. This approach may have preferentially included individuals with greater interest in oral health or dental esthetics, as well as those with easier access to digital platforms, while underrepresenting other population groups. Such selection bias may limit the representativeness of the sample and should be considered when interpreting the findings.

Finally, the assessment of orthodontic treatment need was based exclusively on self-reported perception rather than objective clinical evaluation. While this approach aligns with the psychosocial framework of the study, it does not allow direct comparison with clinically assessed treatment need.

Despite these limitations, the study provides valuable insight into psychosocial and demographic factors associated with smile perception and perceived orthodontic treatment need, highlighting the relevance of patient-centered perspectives in orthodontic care.

## 5. Conclusions

This study identified statistically significant associations between demographic characteristics, self-perception of smile, and perceived orthodontic treatment need. Gender and age were associated with differences in smile satisfaction and awareness of dental esthetics, although effect sizes were generally small to moderate. Perception-related variables, such as behavioral avoidance and dissatisfaction with dental appearance, were also associated with higher perceived treatment need.

The results reflect subjective perceptions within the studied population rather than objective determinants of orthodontic treatment demand.

### 5.1. Implications

From a clinical perspective, the results highlight the relevance of psychosocial factors in patients’ perception of orthodontic needs. Understanding how individuals perceive their smile and dental esthetics may support more patient-centered communication and shared decision-making in orthodontic practice.

### 5.2. Limitations

The main limitations include the cross-sectional design, reliance on self-reported measures, absence of objective clinical assessment, potential selection bias related to online data collection, and limited generalizability due to the regional and demographic composition of the sample.

### 5.3. Future Directions

Future research should adopt longitudinal or mixed-method designs combining self-perception measures with clinical orthodontic indices. Studies involving larger and more diverse populations would improve external validity and help clarify the temporal and causal relationships between psychosocial factors and orthodontic treatment behavior.

Nevertheless, this study also presents important strengths. It provides insight into patients’ self-perception of smile esthetics and perceived orthodontic treatment need using a comprehensive set of psychosocial and awareness-related variables. The use of validated scales and advanced statistical analyses, including correlation analysis and multinomial logistic regression, allowed for a nuanced evaluation of factors influencing perceived treatment need from the patients’ perspective. Moreover, the inclusion of demographic variables contributes to a better understanding of how age, gender, and background shape smile perception and treatment awareness.

## Figures and Tables

**Table 1:** Demographic characteristics of the study sample (*n* = 303).

Variable	Category	*n*	%
*Gender*	Female	195	64.36
	Male	108	35.64
*Age group (years)*	10–20	67	21.84
	21–35	122	40.27
	>35	114	37.62
*Background*	Urban	207	68.32
	Rural	96	31.68

**Table 2:** Self-perception and smile-related behaviors.

Variable	Category	*n*	%
Satisfaction with smile	Very satisfied	54	17.82
	Satisfied	170	56.11
	Somewhat satisfied	49	16.17
	Indifferent	18	5.94
	Dissatisfied	12	3.96
Avoid smiling because of teeth appearance	Never	194	64.03
	Sometimes	95	31.35
	Often/Always	14	4.62
Cover mouth when speaking	Never	241	79.54
	Sometimes	58	19.14
	Often/Always	4	1.32

**Table 3:** Association between gender and smile-related perceptions and awareness (Chi-square analysis).

Variable Analyzed	Test Statistic (χ^2^)	df	*p*-Value	Effect Size (Cramer’s V/Phi)	Interpretation
Level of satisfaction with smile	19.532	4	0.001	Cramer’s V = 0.25–0.30	Statistically significant association; females reported higher satisfaction levels
Concern about the appearance of teeth	25.639	2	<0.001	Phi = 0.291 Cramer’s V = 0.291	Statistically significant association; females showed greater concern about dental appearance

**Table 4:** Significant correlations between age and perception variables.

Variable	Coefficient Type	Coefficient	*p* (Significance)	Number of Respondents (*N*)
Smiling improves mood	Kendall’s tau_b	0.109 *	0.037	303
You cover your mouth when speaking	Kendall’s tau_b	−0.108 *	0.044	303
Smiling improves mood	Spearman’s rho	0.120 *	0.037	303
You cover your mouth when speaking	Spearman’s rho	−0.116 *	0.043	303
Awareness of the importance of straight teeth	Kendall’s tau_b	0.120 *	0.021	303
Need for orthodontic treatment	Kendall’s tau_b	−0.111 *	0.029	303
Awareness of the importance of straight teeth	Spearman’s rho	0.134 *	0.019	303
Need for orthodontic treatment	Spearman’s rho	−0.122 *	0.033	303

* Significant correlation at the 0.05 level (2-tailed).

**Table 5:** Correlation between the need for treatment based on perception and knowledge.

Question Analyzed	Kendall’s tau_b Coefficient	*p*-Value (Significance 2-Tailed)	Number of Respondents (*N*)
How satisfied are you with your smile?	−0.200 **	0.000	303
How concerned are you about the appearance of your teeth?	−0.129 *	0.017	303
Do you try to avoid smiling because of the appearance of your teeth?	0.212 **	0.000	303
Do you ever cover your mouth when speaking?	0.122 **	0.025	303
Do you think your smile would be more attractive if you had your teeth straightened?	0.154 **	0.004	303

* Significant correlation at the 0.05 level (2-tailed). ** Significant correlation at the 0.01 level (2-tailed).

**Table 6:** Summary of factors associated with perceived orthodontic treatment need (multinomial logistic regression).

Predictor Variable	Comparison Category	Direction of Association	Strength of Association	Statistical Significance
Satisfaction with smile	Lower vs. higher satisfaction	Higher perceived need	Small–moderate	*p* < 0.001
Avoidance of smiling	More frequent vs. never	Higher perceived need	Moderate	*p* < 0.001
Covering mouth when speaking	More frequent vs. never	Higher perceived need	Weak	*p* < 0.05
Belief smile would improve with straight teeth	Yes vs. no	Higher perceived need	Moderate	*p* < 0.01
Awareness of importance of straight teeth	Higher vs. lower awareness	Higher perceived need	Weak	*p* < 0.05

## Data Availability

Data available on request from the authors.

## Supplementary Materials

The following supporting information can be downloaded at: https://www.mdpi.com/article/10.3390/jcm15041347/s1. Table S1. English version of the study questionnaire.
