# Supplementary material for: Association Between Psychosocial Factors and the Need for Orthodontic Treatment Based on Self-Perception
Source: J Clin Med. 2026 Feb 9;15(4):1347. doi: 10.3390/jcm15041347 (PMC12942320; doi:10.3390/jcm15041347)
Supplement: Supplementary file 1 [file jcm-15-01347-s001.zip › jcm-4112641-supplementary.pdf]

Table S1. English version of the study questionnaire

I. Patient data

1) Age:

☐6-12 years
☐13-20 years
☐21-35 years
☐over 35 years old

2) Gender:

☐Female
☐Male

3) Background:

☐Urban
☐Rural

II. Questions about self-perception

4) How concerned are you about the appearance of your teeth?

☐Not concerned

☐Somewhat concerned

☐Very concerned

5) How satisfied are you with your smile?

☐Very satisfied

☐Satisfied

☐Indifferent

☐Somewhat satisfied

☐Dissatisfied

6) On a scale of 1 to 5, how much do you think a beautiful smile can improve your mood and self-confidence? (1- minimal awareness; 5- maximum awareness) _______

7) Do you try to avoid smiling because of the appearance of your teeth?

☐Never

☐Sometimes

☐All the time

8) Do you ever cover your mouth when speaking?

☐Never

☐Sometimes

☐All the time

III. Questions related to knowledge and awareness of the need for orthodontic treatment

9) Do you think that straight teeth are important for facial appearance?

☐Yes
☐No
☐I don't know

10) Do you think your smile would be more beautiful if you had your teeth straightened?

☐Yes
☐No
☐I don't know

11) Do you think you need orthodontic treatment?

☐Yes
☐No
☐I don't know
